# Expression of Markers of Endometrial Receptivity in Obese Infertile PCOS Women before and after the Weight Loss Program—A Preliminary Study

**DOI:** 10.3390/cells12010164

**Published:** 2022-12-30

**Authors:** Gaber Bergant, Dzhamilyat Abdulkhalikova, Ana Šuštaršič, Borut Peterlin, Eda Vrtačnik Bokal, Aleš Maver, Mateja Videmšek, Tanja Burnik Papler

**Affiliations:** 1 Clinical Institute of Medical Genetics, University Medical Centre Ljubljana, 1000 Ljubljana, Slovenia; 2 Department of Human Reproduction, Division of Obstetrics and Gynecology, University Medical Centre, 1000 Ljubljana, Slovenia; 3 Faculty of Sports, University of Ljubljana, 1000 Ljubljana, Slovenia; 4 Faculty of Medicine, University of Ljubljana, 1000 Ljubljana, Slovenia

**Keywords:** PCOS, obesity, weight loss, endometrial receptivity, biomarkers

## Abstract

Obesity is an increasing worldwide problem, and it is common in women with polycystic ovaries syndrome (PCOS). It is well known that women with PCOS have lower chances of spontaneous conception as well as lower success with IVF procedures. The mechanisms by which obesity causes lower fertility are not yet fully understood. The aim of the present study was to determine the effect of a lifestyle intervention weight loss program on the expression of the endometrial genes during the window of implantation (WOI). For this purpose, 15 infertile women with obesity and PCOS were included in the study. Endometrial samples were taken during the WOI before and at the end of the program, and RNASeq analysis was performed. There were no significantly differentially expressed genes before and after the weight loss program. We then compared the results of our study with previously published studies on markers of endometrial receptivity. The biomarker genes that were found to be down-regulated during the WOI in previous studies were more down-regulated after the weight loss program in the present study. Furthermore, 25% of the women who achieved the desired 5% or more weight reduction conceived spontaneously. Our study shows that weight loss might positively impact endometrial receptivity. which may lead towards the improved fertility of obese women with PCOS.

## 1. Introduction

Polycystic ovary syndrome (PCOS) is an endocrine disorder that affects between 6 and 20% of reproductive age women [1]. According to the Rotterdam criteria [2], the diagnosis of PCOS is made when two out of three criteria are met: (1) oligo- and/or anovulation; (2) clinical and/or biochemical signs of hyperandrogenism; and (3) polycystic ovaries seen on ultrasound. PCOS is also the most common cause of anovulatory infertility [3]. In addition, many women with PCOS are overweight (body mass index—BMI > 25 kg/m^2^) or obese (BMI > 30 kg/m^2^) [1,4].

In 2008, the ESHRE/ASRM-Sponsored PCOS Consensus Workshop Group decided that the best course of treatment for infertile overweight and obese women with PCOS was weight loss through lifestyle modification. [5]. Even a 5% weight loss improves reproductive functions [6,7] as well as the metabolic and hormonal profile [8] in these women.

Obesity has a negative impact on fertility regardless of the mode of conception, and obese women have an increased chance of anovulatory infertility [9].

Additionally, ovulatory women with regular menstrual cycles who have an increased BMI exhibit a progressive reduction in spontaneous conception with the increase in BMI [10,11]. This indicates that besides ovulation disorders, other factors such as the oocyte, the embryo, or the endometrium are altered in women with an increased BMI [10]. Studies where good quality oocytes from non-obese women were used for oocyte donation have shown that the ongoing pregnancy rate was significantly lower among obese recipients as compared to the lean controls [12,13]. This finding shows that the endometrium has an important role in determining the reproductive outcome.

When comparing the receptive endometrium of lean and obese women, the endometrial gene expression in obese women differed from that of the lean controls. The difference was even more pronounced when obesity was associated with PCOS [14]. A comparison of the enriched biological pathways in the receptive endometrium of overweight and obese women has shown that women who do not conceive in IVF procedures have an over-expression of the pathways related to immune response, inflammation, and reactive oxygen species production [15]. Abdulkhalikova et al. [16] have shown that weight reduction with lifestyle intervention in PCOS women positively affects endometrial receptivity. They have detected changes in the protein abundance of the proteins related to endometrial receptivity after the weight loss program. 

Synchronization between the embryo and the endometrium is crucial for successful embryo implantation. Embryo implantation occurs during the WOI, which is limited to 1 to 2 days of the menstrual cycle. With the emergence of omics, hundreds of simultaneously up- and down-regulated genes expressed in the human endometrium have been proposed as markers of endometrial receptivity [17,18]. In fact, Díaz-Gimeno et al. [17] developed a customized endometrial receptivity array that can be used for the objective evaluation of the endometrial receptivity status in individual women. The downside of transcriptomic studies of endometrial receptivity is that there is not a lot of overlap of the biomarkers between the studies. For this reason, Altmäe et al. [19] performed a meta-analysis of the proposed biomarkers of endometrial receptivity in order to identify a consensus signature of biomarkers. 

The aim of the present study was to determine whether weight reduction influences the expression of the proposed biomarkers of endometrial receptivity in infertile obese women with PCOS. For this purpose, we analyzed the endometrium obtained from obese PCOS women during the WOI before and after the weight loss program.

## 2. Materials and Methods

### 2.1. Study Population

Fifteen (15) infertile PCOS women with obesity were included in the study. The inclusion criteria were: BMI ≥ 30 kg/m^2^, age ≤ 38 years, PCOS diagnosed in accordance with revised Rotterdam criteria [2], primary or secondary infertility, normal partner’s semen, and no other identifiable cause of infertility 

All patients signed a written informed consent form before entering the study. The study was conducted at a tertiary care university hospital in accordance with the Declaration of Helsinki. The study was approved by the National Medical Ethics Committee of the Republic of Slovenia (approval number 0120-491-2017).

### 2.2. Weight Loss Program

Prior to the beginning of the lifestyle intervention program, we performed an evaluation of the anthropometric (body weight, body height, BMI, and proportion of visceral fat) and endocrine-metabolic characteristics (homeostatic insulin resistance model (HOMA-IR), fasting and postprandial glucose and insulin levels, free testosterone, free androgen index (FAI), androstenedione, and dehydroepiandrosterone (DHEA)). We also performed a standard 75 g oral glucose tolerance test (OGTT).

The program was performed as previously described [20]. Briefly, the participants were supposed to attend an 8-week weight loss program prior to inclusion in IVF procedures. After the first 6 weeks, Slovenia went under a complete lockdown due to the COVID-19 pandemic. For this reason, the program was taken online and prolonged for 6 weeks. Altogether, the weight loss program lasted for 12 consecutive weeks. 

The participants took part in group training sessions twice a week for 60 min, beginning six weeks prior to lockdown due to the COVID-19 pandemic (12 training sessions altogether). The intensity of the workout was between 60 and 80% of the maximum heart rate. Most of the exercise units were created as interval training with short rest periods. Additionally, the participants engaged in 30 min of individual aerobic exercise twice a week, including walking, brisk walking, climbing hills, cycling, and running.

After 6 weeks, the group training sessions were modified and held online due to the lockdown. For six more weeks, they were held twice a week for 60 min (12 online training sessions). Every week, the training was given to the participants in a written form. The exercises were created such that they could be carried out with household items or makeshift equipment. Each exercise was illustrated. A brief video demonstrating the fundamentals of each exercise as well as its harder and easier variations was provided. If the participants chose not to conduct the training as stated, they could substitute it with an outdoor aerobic exercise.

A lecture on healthy lifestyle, macro- and micronutrients, and the fundamentals of healthy eating was held at the start of the program. Dietary guidelines were developed with the goal of reducing daily calorie intake by about 250–300 kCal. The participants were advised to keep a diary of consumed food and drinks. The trainer checked the food diary and advised them on how to change the diet if needed.

### 2.3. Endometrial Biopsy

Endometrial biopsies were performed in all the patients. However, only the endometrial samples of the women who reduced their BMIs by 5% or more were used for RNA sequencing analyses. All the women had regular menstrual cycles; therefore, the endometrial biopsy was performed using Rampipella (RI.MOS., Mirandola, Italy) between the 21st and the 23rd day of the menstrual cycle. Biopsies were performed before the start and after the end of the weight loss program. The samples were immediately frozen in liquid nitrogen and kept at 80 °C until processing.

### 2.4. RNA Isolation and RNA Sequencing

Total RNA was isolated from the endometrial samples using the Qiagen RNeasy kit (Qiagen, Hilden, Germany) according to the manufacturer’s protocol. The samples were then sequenced using paired-end sequencing on the Illumina NextSeq 550 in 75 cycles to an average depth of 2.75 million reads per sample. All the samples were processed from RNA isolation to sequencing as a single batch. Next, expression quantification was performed with salmon in a quasi-mapping mode using the Ensembl human GRCh38 transcript atlas, followed by transcript-level quantification aggregation to gene level using the tximport R package [21,22]. Quality control was performed in the R programing environment with principal component analysis (PCA) and unsupervised clustering with heatmap plot inspection.

### 2.5. Selection of Target Genes

Gene targets were obtained from previously published studies on the transcriptomic signature of human endometrial receptivity by Diaz-Gimeno et al. [17] and Altmäe et al. [19]. In order to discover a meta-signature of endometrial receptivity, the authors of the second study conducted a meta-analysis of the endometrial receptivity-associated genes on 164 samples from the pre-receptive and mid-secretory phases of the endometrium. Fifty-seven (57) genes were identified as endometrial receptivity markers, with 39 experimentally confirmed using RNA sequencing on two additional datasets. Of the 57 genes, 52 were up-regulated and 5 were down-regulated when comparing the pre-receptive and mid-secretory phases of the endometrium. In the study by Díaz-Gimeno et al. [17], gene expression in the receptive endometrium was performed and compared with the pre-receptive and proliferative phases of the endometrium using a customized endometrial receptivity microarray to define the transcriptomic signature of the receptive human endometrium. In total, 238 genes were found to be differentially expressed between the receptive and non-receptive groups, with 143 up-regulated genes and 95 down-regulated genes.

### 2.6. Statistical Analysis

The IBM SPSS Statistics for Windows, Version 25.0. (IBM Corp.: Armonk, NY, USA) was used to perform the statistical analysis. The values were considered significant at *p* < 0.05.

The differences in the measured variables before and after the weight loss program were estimated using a paired t test. The numerical data are presented as a mean with standard deviation if normally distributed or as a median with 1st and 3rd quartile if skewed.

Differential gene expression was performed using the DESeq2 bioconductor R package and the ashr shrinkage estimator to reveal possible differentially expressed genes between the samples obtained prior to losing weight and following the weight loss in the obese women [23,24]. Additionally, we performed gene set enrichment analysis (GSEA) using the fgsea bioconductor R package [22] and gene sets constructed from previously published studies [17,19]. The gene sets used in the analysis are presented in Supplementary Table S1. The Fgsea function number of the permutations parameter used in the GSEA was set to 1000. The other parameters were left under their default settings.

## 3. Results

### 3.1. Anthropometric and Endocrine Parameters

On average, BMI was 3.7% lower after the end of the weight loss program and 12 of the 15 women (75%) achieved the desired BMI reduction of 5% or more. Free testosterone levels were significantly lower at the end of the program, whereas the other measured parameters did not differ significantly. Table 1 and Table 2 show the differences in the anthropometric and metabolic parameters before and after the weight loss program.

### 3.2. Spontaneous Pregnancy after the Weight Loss Program

All of the participants in the current study wanted to conceive and planned to be included in IVF when the program was finished. However, in the three months following the program’s conclusion, 3 of the 15 participating women (20%) spontaneously conceived. All of them achieved the desired 5% BMI reduction. When calculating the spontaneous pregnancy rate only for the women who achieved the desired BMI reduction, the rate was even higher (25%).

### 3.3. Biomarker Expression Analysis

Of the 15 included women, 11 endometrial samples obtained before the beginning of the weight loss program and 9 samples obtained after the end of the weight loss program were used for analyses. For the post-weight loss RNA Seq analyses, only the endometrial samples of the women who reduced their BMI by 5% or more were considered. The unsupervised clustering analysis revealed three separate sample sets which could not be attributed to any of the known sample parameters. For this reason, further gene expression analyses were corrected for an additional source of variation, likely introduced into the experiment due to the narrow WOI and the associated sample procurement challenges.

DESeq2 analysis was performed on the 248 unique endometrial receptivity genes reported in the previous studies [17,19]. None of the genes surpassed the statistical significance threshold following the Benjamini–Hochberg correction for multiple testing.

We additionally performed a GSEA using gene sets of endometrial receptivity from the two previously published studies, separately [17,19] as well as via the union of the reported up- and down-regulated genes from both studies. The GSEA showed statistically significant differences in gene expression in the two analyzed sets. First, the down-regulated genes obtained from the union of 96 genes reported to be down-regulated in the receptive endometrium in the previous two studies were also significantly down-regulated in the endometrial samples obtained after the end of the weight loss program in our study. Second, the genes that were shown to be down-regulated in the receptive endometrium reported by Altmäe et al. [19] were also significantly down-regulated in the endometrial samples obtained after the end of the weight loss program in the present study.

In the current investigation, there was no significant difference in the expression of the gene sets that were up-regulated in the studies by Altmäe et al. [19] and Díaz-Gimeno et al. [17] following the conclusion of the weight loss program.

## 4. Discussion

This is the first report on the effect of weight reduction on the expression of the previously reported genetic markers of endometrial receptivity in obese PCOS women.

The results of the studies where the influence of obesity and weight loss on female fertility was observed are contradictory. Legro et al. [25] included 379 obese women with unexplained infertility in a ratio of 1:1 in a 16-week program. The women in the first study group had an intensive weight loss program with increased physical activity, meal replacements, and medication. The second group of women had increased physical activity alone without weight loss. The women who did not become pregnant spontaneously at the end of the program were given up to three rounds of ovarian stimulation/intrauterine insemination. In terms of live birth rates, there were no appreciable differences between the groups. They concluded that weight loss prior to infertility treatment is not beneficial for obese women with unexplained infertility. However, the same research team also found that delayed fertility therapy combined with preconception weight loss was superior to immediate therapy for obese PCOS women [26]. They compared ovulation and live birth rates in women who immediately received clomiphene citrate (CC) for ovulation induction and women who first underwent weight reduction with lifestyle intervention and then received CC. They reported significantly higher ovulation (44.7% vs. 62%) and live birth rates (10.2% vs. 25%) in the group who first attended the weight loss program. Furthermore, other studies have also shown a negative impact of obesity and a beneficial effect of weight loss on fertility and IVF outcomes [27,28]. A meta-analysis by Sermondade et al. [27] has shown a decreased probability of LBR after IVF procedures in obese women when compared with women of normal weight. The chances of live birth were even lower when obesity was associated with PCOS. Furthermore, a meta-analysis by Best et al. [28] has shown that weight loss interventions with diet and exercise improve pregnancy rates. In line with this research, the current study demonstrates that weight loss in obese infertile women with PCOS may improve the likelihood of spontaneous conception as 25% of the women who reduced their body weight by at least 5% became pregnant on their own within the first three months after finishing the weight loss program.

The mechanisms of how obesity impairs fertility are not yet completely understood. There is increasing evidence that women with PCOS and obesity have an impaired endometrial functioning [14,29]. Our study aimed to determine the potential change in the endometrial gene expression profile during the WOI after the weight loss program. As there were no significantly differentially expressed genes, we decided to compare our results with previously published studies on the markers of endometrial receptivity. Since numerous studies have published hundreds of different genes as markers of endometrial receptivity with very little overlap between the studies, we decided to analyze the expression of genes that were obtained in a meta-analysis of nine studies [19] as well as those included in a commercial microarray used for the determination of the WOI [17]. To increase the power of the study, we merged the list of differentially expressed markers from both studies. Using GSEA, we were able to partially replicate the direction of expression change towards the receptive state of the endometrium. After the weight loss program, an increased down-regulation of the genes that were recognized as markers of the receptive endometrium and were down-regulated during the WOI in previous studies [17,19] was seen. As all women in the present study had regular menstrual cycles and the weight loss program was the only intervention, we presume that a shift towards a more receptive endometrium occurred due to weight reduction.

Lastly, there was a significant decrease in free testosterone levels after the weight loss program. This finding is in accordance with previous studies which report on the decrease in androgen levels after weight loss [30]. Studies have shown that higher serum testosterone levels correlate negatively with fertilization rates in IVF procedures [31]. The lowering of serum testosterone levels after the weight loss program may therefore positively influence the success of IVF procedures.

## 5. Conclusions

To the best of our knowledge, this study is the first to document the alteration in endometrial gene expression during the WOI in obese infertile PCOS women following the weight loss program. Moreover, for the first time, a comparison with previously published markers of endometrial receptivity after the weight loss program was performed. Collectively, the results of the present study imply that weight reduction in obese PCOS infertile women might positively affect endometrial receptivity and is therefore the right approach for treating these women. We have shown that the genes that were proposed as biomarkers of endometrial receptivity and are down-regulated in the receptive endometrium are more down-regulated in the endometria of obese PCOS women after the successful weight loss (5% or more).

Furthermore, spontaneous conception occurred in 25% of the women whose weight loss program was successful (i.e., 5% or more weight reduction). Women who conceived spontaneously were spared of IVF procedures, which represent a significant psychological, physical, and financial burden. We believe that this finding will have a significant impact on future counselling as well as on the treatment of infertile PCOS women with obesity.

The limitation of the present study is the relatively small number of endometrial samples analyzed. However, one must consider that the group of women included in the study was highly specific and when all the inclusion criteria were considered not many women remained to be included in the study. Furthermore, not all the women who met the criteria wanted to be included in the study. Once they began with the weight loss program, they needed constant motivation to finish it. The motivation was even harder to achieve once the lockdown due to the COVID-19 pandemic started and the program was taken online. We therefore consider the fact that 75% of the included women achieved the desired 5% weight reduction to be a great success. Furthermore, although not all the women achieved the 5% reduction, all the women lost weight and changed their lifestyle habits, which will have a positive effect on their future health.

## Figures and Tables

**Table 1:** Anthropometric parameters before and after the weight loss.

	Before	After	*p*-Value
Body weight (kg)	103.9 ± 15.1	98.5 ± 14.9	<0.001
BMI (kg/m^2^)	36.1 ± 5.1	34.3 ± 5.0	<0.001
Waist circumference (cm)	106.7 ± 9.7	99.8 ± 9.9	<0.001
Abdominal circumference (cm)	120.0 ± 12.8	111.7 ± 13.2	<0.001
Hip circumference (cm)	125.8 ± 10.0	120.7 ± 9.2	<0.001

Values are depicted as mean ± SD.

**Table 2:** Metabolic and endocrine parameters before and after the weight loss.

	Before (N = 12)	After (N = 12)	*p*-Value
Fasting glucose (mmol/L)	5.63 ± 0.53	5.41 ± 0.33	NS
Fasting insulin (pmol/L)	19.6 ± 9.0	14.2 ± 6.1	NS
HOMA-IR	4.98 ± 2.47	3.44 ± 1.62	NS
Free testosterone (pmol/L)	6.59 ± 2.05	5 ± 1.2	**0.01**
SHBG (nmol/L)	42.9 ± 20.2	46.6 ± 26.6	NS
DHEAS (µmol/L)	6.8 ± 2.3	5.9 ± 1.9	NS
FAI	3.3 ± 1.7	3.3 ± 2	NS
Androstenedione (nmol/L)	6.68 ± 3.21	6.75 ± 2.71	NS

Values are depicted as mean ± SD. HOMA-IR—homeostatic model assessment for insulin resistance; SHBG—sex hormone binding globulin; DHEAS—dehydroepiandrosterone sulfate.

## Data Availability

All data are available from the authors upon request.

## Supplementary Materials

The following supporting information can be downloaded at: https://www.mdpi.com/article/10.3390/cells12010164/s1, Table S1: gene sets used for GSEA.
