# Supplementary material for: Expression of Markers of Endometrial Receptivity in Obese Infertile PCOS Women before and after the Weight Loss Program—A Preliminary Study
Source: Cells. 2022 Dec 30;12(1):164. doi: 10.3390/cells12010164 (PMC9818464; doi:10.3390/cells12010164)
Supplement: Supplementary file 1 [file cells-12-00164-s001.zip › cells-2103734-supplementary.pdf]

TableS1 Gene sets used for gene set enrichment analysis (.gmt formatting is supported by GSEA algorithm). FC – fold-change.

**Gene set description**	**Gene set content (.gmt formatting)**
**Genes reported by Diaz-Gimeno et al. with a positive FC**	Diaz-Gimeno et al. positive FC > Diaz-Gimeno et al. positive FC PAEP SPP1 GPX3 MAOA GADD45A DKK1 CLDN4 C4BPA IL15 CD55 ID4 GNLY CP SLC1A1 DPP4 TCN1 CFD ANXA4 EFNA1 GBP2 APOD BCL6 CEBPD ACADSB DEPP1 ABCC3 MT1G ARG2 COMP G0S2 TSPAN8 DEFB1 MAP3K5 EDNRB PRUNE2 S100P IGFBP1 DDX52 SERPING1 ARID5B LAMB3 AOX1 IDO1 ANXA2 HABP2 C1R AQP3 DYNLT3 MT1H NNMT NDRG1 ENPEP
**Genes reported by Diaz-Gimeno et al. with a negative FC**	Diaz-Gimeno et al. negative FC > Diaz-Gimeno et al. negative FC SFRP4 EDN3 OLFM1 CRABP2 MMP7
**Genes reported by Altmäe et al. with a positive FC**	Altmäe et al. positive FC > Altmäe et al. positive FC GPX3 PAEP COMP SLC1A1 LIF TCN1 CXCL14 C4BPA TSPAN8 LAMB3 MAOA SOD2 GADD45A MUC16 THBD NNMT DPP4 SCGB2A2 S100P SNX10 CP G0S2 LYPD3 ANG ABCC3 XCL1 ADRA2A EFNA1 KLRC1 TAGLN SLC15A1 IGFBP1 PTGER2 THBS2 HPSE SERPING1 CFD CRISP3 CATSPERB RPRM GAST EDNRB RARRES1 ABLIM3 GLIPR1 AOX1 CYP3A5 CTSW DHRS3 MYL9 CLUIER3 GPRC5C C3 AQP3 S100A4 GNLY IDO1 NKG7 CORO1A SLPI ARG2 ANXA4 ARID5B DEFB1 SPP1 DKK1 HABP2 GZMA TBC1D2 CDA DEPP1 SLC38A1 FLJ20152 STAR LMOD1 HAND2 IMPA2 CXCL13 PNP DEPTOR RARRES3 XCL2 MT2A TIMP3 RRAS CLDN10 AGR2 PROM1 COTL1 ACTA2 ID4 FAM59A GAS1 FGB LMCD1 SYNE2 TRA MRPS2 KCNK7 KRT7 GDF15 MFAP5 IGF2 MT1G VCAM1 CLDN4 PTPRR AIMP1 DDX52 HAL P3H2 ADAMTS1 TH PROS1 EMCN FOSL2 PSMB10 ACADSB S100A1 AMIGO2 PLA2G16 ARHGAP45 POLD4 CD7 ARHGDIB LRFN4 GBP2 GABARAPL1 MT1H FXYD2 RNASE4 PPARGC1A CYBRD1 ENPEP CES1 ATP6V1A ASS1 IL15 BCL6 EVC IL2RB EFEMP1
**Genes reported by Altmäe et al. with a negative FC**	Altmäe et al. negative FC > Altmäe et al. negative FC TRH HLA-DOB ATP6V0E2 CSRP2 OLFM4 SLC15A2

	CALB2 SFRP4 CTNNA2 NR4A2 DUOX1 KIF20A PENK POSTN LRP4 SLC16A6ADGRG2 RANBP17 EDN3 CEP55 CENPE FAM169A COL16A1 GALNT12 ANK3 CAPN6 HPGD SLC7A1 MMP26 LRRC17 KCNG1 CYP2J2 MFAP2 ALPL CDK10 GREM2 SOX17 TACC3 HEY2 PAQR4 PRR15L HSD11B2 KIF11 NRG2 OLFM1 COBL TOP2A NDC80 MTCL1 SERPINA5 DLGAP5 ASPM SPDEF KMO CREB3L1 ATP1B1 ECI2 IDH1 CBR3 MPPED2 CKB ADAMTS8 BIRC3 WHRN KHDRBS3 GALNT4 PMEPA1 KIF4A KCNJ2 BARD1 MAP2K6 OFD1 PLA1A RAD54B PBK NDRG2 ECM1 PRC1 MSX1 CCNB2 LRRC1 SORD EPHB3 TMSB15A RASSF2 TTC21B OPRK1 ANO1 CRABP2 FANCI PRKCQ CDK1 BUB1B STEAP4 HEY1
**Genes with a positive FC reported by either Diaz-Gimeno et al. or Altmäe et al.**	OVERLY_EXPRESSED_GENES > Diaz-Gimeno et al. positive FC and Altmäe et al. positive FC ABCC3 ABLIM3 ACADSB ACTA2 ADAMTS1 ADRA2A AGR2 AIMP1 AMIGO2 ANG ANXA2 ANXA4 AOX1 APOD AQP3 ARG2 ARHGAP45 ARHGDIB ARID5B ASS1 ATP6V1A BCL6 C1R C3 C4BPA CATSPERB CD55 CD7 CDA CEBPD CES1 CFD CLDN10 CLDN4 CLU COMP CORO1A COTL1 CP CRISP3 CTSW CXCL13 CXCL14 CYBRD1 CYP3A5 DDX52 DEFB1 DEPP1 DEPTOR DHRS3 DKK1 DPP4 DYNLT3 EDNRB EFEMP1 EFNA1 EMCN ENPEP EVC FAM59A FGB FLJ20152 FOSL2 FXYD2 G0S2 GABARAPL1 GADD45A GAS1 GAST GBP2 GDF15 GLIPR1 GNLY GPRC5C GPX3 GZMA HABP2 HAL HAND2 HPSE ID4 IDO1 IER3 IGF2 IGFBP1 IL15 IL2RB IMPA2 KCNK7 KLRC1 KRT7 LAMB3 LIF LMCD1 LMOD1 LRFN4 LYPD3 MAOA MAP3K5 MFAP5 MRPS2 MT1G MT1H MT2A MUC16 MYL9 NDRG1 NKG7 NNMT P3H2 PAEP PLA2G16 PNP POLD4 PPARGC1A PROM1 PROS1 PRUNE2 PSMB10 PTGER2 PTPRR RARRES1 RARRES3 RNASE4 RPRM RRAS S100A1 S100A4 S100P SCGB2A2 SERPING1 SLC15A1 SLC1A1 SLC38A1 SLPI SNX10 SOD2 SPP1 STAR SYNE2 TAGLN TBC1D2 TCN1 TH THBD THBS2 TIMP3 TRATSPAN8 VCAM1 XCL1 XCL2

**Genes with a negative FC reported by either Diaz-Gimeno et al. or Altmäe et al.**	UNDER_EXPRESSED_GENES > Diaz-Gimeno et al. negative FC and Altmäe et al. negative FC ADAMTS8 ADGRG2 ALPL ANK3 ANO1 ASPM ATP1B1 ATP6V0E2 BARD1 BIRC3 BUB1B CALB2 CAPN6 CBR3 CCNB2 CDK1 CDK10 CENPE CEP55 CKB COBL COL16A1 CRABP2 CREB3L1 CSRP2 CTNNA2 CYP2J2 DLGAP5 DUOX1 ECI2 ECM1 EDN3 EPHB3 FAM169A FANCI GALNT12 GALNT4 GREM2 HEY1 HEY2 HLA-DOB HPGD HSD11B2 IDH1 KCNG1 KCNJ2 KHDRBS3 KIF11 KIF20A KIF4A KMO LRP4 LRRC1 LRRC17 MAP2K6 MFAP2 MMP26 MMP7 MPPED2 MSX1 MTCL1 NDC80 NDRG2 NR4A2 NRG2 OFD1 OLFM1 OLFM4 OPRK1 PAQR4 PBK PENK PLA1A PMEPA1 POSTN PRC1 PRKCQ PRR15L RAD54B RANBP17 RASSF2 SERPINA5 SFRP4 SLC15A2 SLC16A6 SLC7A1 SORD SOX17 SPDEF STEAP4 TACC3 TMSB15A TOP2A TRH TTC21B WHRN
